# Lack of Association between Common Polymorphisms in Selenoprotein P Gene and Susceptibility to Colorectal Cancer, Breast Cancer, and Prostate Cancer: A Meta-Analysis

**DOI:** 10.1155/2021/6525449

**Published:** 2021-09-27

**Authors:** Hanjiang Xu, Fan Mo, Jun Zhou, Zongyao Hao, Xianguo Chen, Chaozhao Liang

**Affiliations:** ^1^Department of Urology, The First Affiliated Hospital of Anhui Medical University, Hefei 230000, China; ^2^Institute of Urology, Anhui Medical University, Hefei 230000, China; ^3^Anhui Province Key Laboratory of Genitourinary Diseases, Anhui Medical University, Hefei 230000, China

## Abstract

**Method:**

We search the PubMed, Embase, Google Scholar, and Wanfang (China) databases (up to December 1, 2020) to identify all eligible publications. The pooled odds ratio (OR) correspondence with 95% confidence interval (CI) was calculated to evaluate the associations.

**Results:**

Finally, nine eligible studies with 7,157 cases and 6,440 controls and five studies with 2,278 cases and 2,821 controls were enrolled in rs3877899 and rs7579 polymorphisms, individually. However, a null significant association was detected between the two polymorphisms in *SEPP1* and susceptibility to colorectal, breast, and prostate cancer in all comparison models. Subsequently, subgroup analysis based on tumor type, no significant association was identified for prostate, breast, and colorectal cancer. In addition, when the stratification analyses were conducted by the source of control, HWE status, and ethnicity, yet no significant association was found.

**Conclusions:**

The current meta-analysis shows that SEPP1 rs3877899 and rs7579 polymorphisms may not be associated with susceptibility to colon cancer, breast cancer, and prostate cancer, and further well-designed studies with a larger sample size are warranted to validate our findings.

## 1. Introduction

There has been a progressive increase in the global incidence of malignancies, causing a serious threat to human health, presently, among the main causes of death [1]. Increasing evidence suggests that cancers are multifactorial diseases, which derive from complex coactions between genetic and environmental factors [2].

Oxidative stress, which causes mitochondrial damage and DNA breakage by reactive oxygen species (ROS), is closely related to tumor progression [3, 4]. ROS, like hydrogen peroxide (H_2_O_2_), can cause DNA damage due to the continuous production of various cellular metabolic processes in the body, which may lead to malignant transformation of cells [5]. Selenoprotein P (SEPP1) is the dominant selenoprotein in plasma as two isoforms (~50 kDa and ~60 kDa) and is believed to have two main functions: providing tissues with selenium for tissues and exerting antioxidant defense capabilities [6]. The insufficiency of SEPP1 may participate in the occurrence and progression of cancer. Earlier studies have testified high expression of SEPP1 in colonic mucosa and relatively lower expression of SEPP1 in human colon tumors. Moreover, SEPP1 affects colitis-induced tumorigenesis through regulating stemness and oxidative damage also has been confirmed in the study [7]. In Calvo et al.'s research, they advanced pointed that the SEPP1 was reduced in prostate cancer (PCa) [8]. Gonzalez-Moreno et al.'s study has shown that knockdown of SEPP1 expression in prostate epithelial neoplasia lesion cell lines and invasive tumors significantly increased ROS and cell growth inhibition after exposure to H_2_O_2_ [9].

Nowadays, more and more studies have demonstrated that several polymorphisms of the selenoprotein P gene (*SEPP1*) were associated with susceptibility of tumors, including breast cancer (BC) [10], colorectal cancer (CRC) [11], and PCa [12]. However, results from these studies remain inconclusive. In order to yield a more accurate and robust estimation, we conducted this meta-analysis trying to comprehensively analyze the connection between two common polymorphisms (rs3877899 and rs7579) in *SEPP1* and cancer susceptibility.

## 2. Materials and Methods

### 2.1. Search Strategy

We conducted this meta-analysis on the basis of the PRISMA meta-analysis guidelines [13]. A comprehensively retrieve of the literature concerning relationships between the *SEPP1* polymorphisms and cancer susceptibility was performed on PubMed, Embase, and Google Scholar databases (up to December 1, 2020) by using the following searching terms: “SEPP1 OR Selenoprotein P” AND “polymorphism OR variation OR SNP OR genotype OR allele OR mutation” AND “cancer OR malignancy OR tumor OR neoplasm OR carcinoma”. We also conducted manual searches on the references of these selected original studies to identify other eligible studies.

### 2.2. Inclusion and Exclusion Criteria

Included literature should be in line with the following criteria: (1) studies that evaluated the relationship between SEPP1 polymorphisms (rs3877899 and rs7579) and cancer susceptibility; (2) sufficient genotype data from the text or the supporting information; (3) case-control studies. Moreover, these studies should also be excluded when they were as follows: (1) insufficient data; (2) not a case-control study, such as Comments, Case Reports, and Reviews; (3) the total scores of Newcastle-Ottawa Scale (NOS) is less than 5 (The quality of the enrolled studies was assessed by NOS (Newcastle-Ottawa Scale), which is presented in Table 1. In addition, the specific scoring rules of NOS are listed in Table S1.).

### 2.3. Data Extraction

Two reviewers (Hanjiang Xu and Fan Mo) have devoted themselves to the data extraction process referring to the predetermined criteria. All the discrepancies were settled through discussion till all consensus was settled. Furthermore, the following details should also be extracted: name of the first author, publication year, source of controls, ethnicity of a case-control study, genotype frequencies of cases and controls, and so on.

### 2.4. Statistical Analysis

We calculated the odds ratio (OR) with 95% CI confidence interval (CI) to appraise the intensity of relationships between *SEPP1* polymorphisms (rs3877899 and rs7579) and cancer susceptibility in the following genetic models: allele contrast (A vs. G), recessive (AA vs. AG+GG), dominant (AA+AG vs. GG), heterozygous (AG vs. GG), and homozygous (AA vs. GG) models (G: wild allele; A: variant allele). We assessed the statistical heterogeneity hypothesis through *I*^2^ statistics to quantify the inconsistency, which represents the proportion of variability between studies that potentially arose from heterogeneity instead of contingency. *I*^2^ values greater than 50% are considered to have significant heterogeneity [14], indicating the random-effects model would be selected to calculate the pooled OR estimated value of individual study; if not, the fixed-effects model was obtained. Our current study also assessed sensitivity analysis as well as publication bias [15]. We use Stata software for all statistical analysis (version 12.0; STATA Corp, College Station, TX). *P* ≤ 0.05 was considered statistically significant.

## 3. Results

### 3.1. Characteristics of Studies

**Figure 1:**
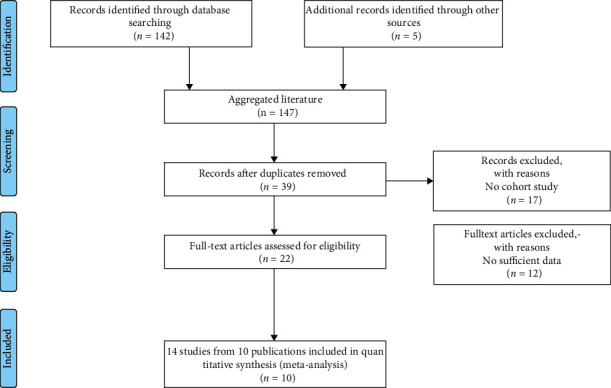
The eligible study selection process.

Overall, 10 publications with 14 independent studies on *SEPP1* polymorphisms (rs3877899 and rs7579) and cancer susceptibility were available for our meta-analysis [10, 11, 16–23], and the publication selection process is displayed in Figure 1. For rs3877899 polymorphism, nine case-control studies with 7,157 cases and 6,440 controls met the inclusion criteria, including three BC, two CRC, and four PCa studies. For rs7579 polymorphism, there were five studies (one BC study, one PCa study, and three CRC studies) with 2,278 cases and 2,821 controls that met the eligibility criteria. Cancers were confirmed pathologically or histologically in most studies. The authors of included studies used a variety of genotyping methods, including PCR-RFLP and TaqMan. We think the earlier sentence was correct regarding the genotyping methods used in the included studies. Except for these studies [10, 11, 20, 23], the genotype distribution in control groups of the enrolled studies was in line with HWE. The selected study characteristics are enumerated in Table 2.

### 3.2. Pooled Analysis

A result of the detailed associations of *SEPP1* polymorphisms with cancer susceptibility in all of the genetic models is presented in Table 3. And the results demonstrated that no evidence of the relevance between the two polymorphisms (rs3877899 and rs7579) and susceptibility to CRC, BC, and PCa was found in each genetic model (Table 3).

For rs3877899 polymorphism, no significant association was found when pooling all the eligible studies (A vs. G: OR = 1.099, 95% CI: 0.938-1.287, *P* = 0.243; AA vs. GG: OR = 1.129, 95% CI: 0.794-1.605, *P* = 0.498; AG vs. GG: OR = 1.035, 95% CI: 0.909-1.179, *P* = 0.603; AA+AG vs. GG: OR = 1.079, 95% CI: 0.919-1.267, *P* = 0.356; AA vs. AG+GG: OR = 1.017, 95% CI: 0.871-1.189, *P* = 0.555, Table 3). In addition, there was also no significant relationship between rs7579 polymorphism and cancer risk in each genetic models (A vs. G: OR = 1.090, 95% CI: 0.923-1.286, *P* = 0.309; AA vs. GG: OR = 1.267, 95% CI: 0.861-1.866, *P* = 0.230; AG vs. GG: OR = 1.022, 95% CI: 0.908-1.151, *P* = 0.715; AA+AG vs. GG: OR = 1.075, 95% CI: 0.897-1.287, *P* = 0.434; AA vs. AG+GG: OR = 1.209, 95% CI: 0.878-1.666, *P* = 0.245, Table 3).

### 3.3. Subgroup Analysis

As to a stratification analysis conducted by cancer type, no association was identified for PCa, BC, and CRC of rs3877899 polymorphism in all five genetic models (Table 3). In addition, we also conducted stratification analyses by the source of control, ethnicity, and HWE status for both two polymorphisms; the null association was detected (Table 3). In all subgroups, the number of included studies is not less than 3 (*n* ≥ 3).

### 3.4. Heterogeneity Evaluation

Table 3 shows that the statistical heterogeneity within studies was evaluated by a chi-squared-based *Q*-statistic test. When *P* > 0.10, the fixed-effects model (the Mantel-Haenszel model) was used; else, the random-effects model (the DerSimonian-Laird model) was adopted.

### 3.5. Sensitivity Analysis and Publication Bias

**Figure 2:**
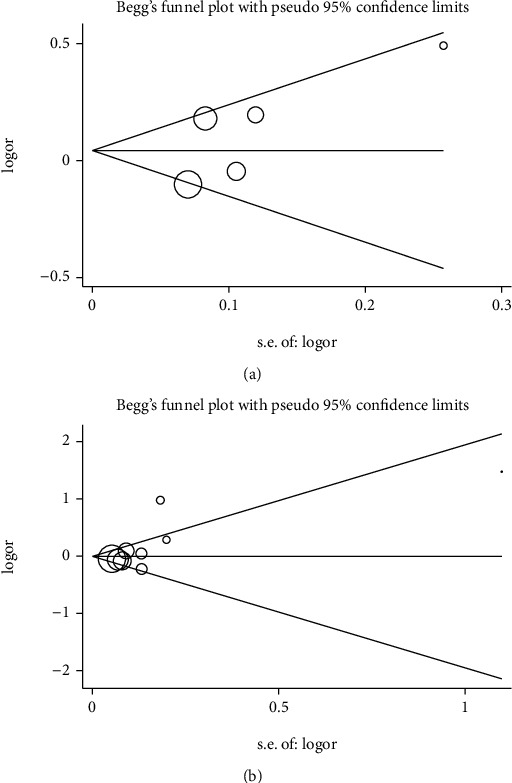
(a) Begg's funnel plot of publication bias (for rs7579 polymorphism, A vs. G). Each point represents a separate study for the indicated association. Log (OR), natural logarithm of OR; horizontal line, mean effect size. CI = confidence interval; OR = odds ratio. (b) Begg's funnel plot of publication bias (for rs3877899 polymorphism, GA vs. GG). Each point represents a separate study for the indicated association. Log (OR), natural logarithm of OR; horizontal line, mean effect size. CI = confidence interval; OR = odds ratio.

We explored the impact of each study on the pooled OR by excluding one study from the pooled analysis, thereby performing a sensitivity analysis. It turns out there is no material influence on the stability of the results. So as to assess the publication bias of the existing literature, we performed Begg's funnel plot as well as Egger's test. In all comparison models, the pattern of the funnel chart was roughly symmetrical (Figures 2(a) and 2(b)). In addition, we also used the Egger test, and these results indicated no publication bias (Table 3).

## 4. Discussion

SEPP1 plays an important role in both supplying selenium to tissues and exerting antioxidant defenses. The delivery of selenium is accomplished by the C-terminal domain of SEPP1, which includes nine selenocysteine residues, while antioxidation is accomplished by selenocysteine that has been shown to have peroxidase activity [24]. The antioxidant function of SEPP1 suggests that it has the effect of preventing cancer, especially in inflammatory cancer characterized by increased oxidative stress [25]. Dysfunction of SEPP1 may contribute to the occurrence and progression of cancer.

The human SEPP gene (SEPP1) contains two functional polymorphisms, rs3877899 (Ala234Thr) and rs7579 (Gram A base mutation in SEPP1 mRNA 3′UTR), which affect the selenoprotein activity of plasma and lymphocytes and the relative proportion of plasma SEPP isotypes in vivo experiments [26, 27]. Therefore, the mutation of *SEPP1* will produce some nonfunctional or low-functional protein subtypes, reducing the antioxidant activity of SEPP1. At the same time, the accumulation of peroxide is conducive to the production and development of cancer. Therefore, mutations in *SEPP1* theoretically increase the susceptibility of tumors.

Both polymorphisms of *SEPP1* have been reported to be related to the risk of PCa [19] and CRC [20]. Furthermore, in the study conducted by Meplan et al. [18], a connection between the *SEPP1* rs3877899 mutation and the risk of BC was also found. However, in a study by Jablonska et al. [22] in Polish women, there was no evidence of a relation between BC risk and the rs3877899 polymorphism. In fact, many related epidemiological studies have been carried out so far, but no definite conclusions have been obtained, and some results are even controversial. Therefore, in order to clarify the relationship between cancer risk and the *SEPP1* polymorphism, we conducted this meta-analysis. After pooling all data from 7,157 cases and 6,440 controls for the rs3877899 polymorphism and 2,278 cases and 2,821 controls for the rs7579 polymorphism, a null significant association was identified between *SEPP1* polymorphism and cancers (prostate, breast, and colorectal cancer) in all comparative models. We subsequently did subgroup analyses based on cancer type control source HWE status and race for both polymorphisms and found no significant association.

This result contradicts our previous theoretical speculation. In fact, *SEPP1* polymorphism affects tumor susceptibility through the antioxidant activity of SEPP1 protein. However, there may be more than one factor that can change the antioxidant activity of SEPP1 protein. The study by Sutherland et al. [23] showed that the two polymorphisms of *SEPP1* (rs3877899 and rs7579) are not associated with the risk of CRC, which may be due to the inconsistent dietary selenium intake of the subjects. It is possible that when the plasma selenium level is low, the difference in the plasma level of SEPP1 caused by genetic polymorphism can be reflected, and this difference will disappear after selenium supplementation [26]. Furthermore, our results can be analyzed more accurately by age, cancer grade, and environmental factors (such as selenium status related to SEPP1 expression). For example, in the study of Cooper et al. [21], cancer was divided into two groups of nonprogressive and progressive, and even the factor of smoking was included. Therefore, to explore the impact of genotype on cancer susceptibility, environmental and nutritional factors should be strictly controlled; otherwise, the results may be biased.

In conclusion, despite providing a sufficient statistical sample size to enhance the reliability of our findings, there are some shortcomings of the study. Firstly, the relatively small number of included studies would be a limitation and may constrain our conclusions. Secondly, we only searched papers published in a limited number of databases and some studies may have been overlooked. Finally, the results may be false negative, because some included studies show a significant relation between *SEPP1* polymorphism and cancer susceptibility.

## 5. Conclusions

In this meta-analysis, our results find no association between *SEPP1* rs3877899 and rs7579 polymorphisms and susceptibility to CRC, BC, and PCa. Taking into account the complex interactions between genes and the environment, it is necessary to conduct unbiased studies with more sample sizes and more cancer types in different ethnic groups.

## Figures and Tables

**Table 1:** Methodological quality of the included studies according to the Newcastle-Ottawa Scale.

Variants	Author	Representativeness of cases	Source of controls	HWE in controls	Genotyping examination	Association assessment	Total scores
rs7579	Meplan et al.	∗∗	∗∗	∗∗	0	∗∗	8
	Steinbrecher et al.	∗∗	∗∗	∗∗	0	∗∗	8
	Meplan et al.	∗∗	∗	∗	0	∗	5
	Sutherland et al.	∗∗	∗	∗	0	∗∗	6
	Amini et al.	∗∗	∗∗	∗	0	∗∗	7

rs3877899	Steinbrecher et al.	∗∗	∗∗	∗∗	0	∗∗	8
	Cooper et al.	∗∗	∗∗	∗∗	0	∗∗	8
	Geybels et al.	∗∗	∗∗	∗∗	0	∗∗	8
	Karunasinghe et al.	∗∗	∗∗	∗∗	0	∗∗	8
	Meplan et al.	∗∗	∗	∗	0	∗	5
	Sutherland et al.	∗∗	∗	∗∗	0	∗∗	7
	Meplan et al.	∗∗	∗∗	∗∗	0	∗∗	8
	Jablonska et al.	∗∗	∗∗	∗∗	0	∗∗	8
	Mohammaddoust et al.	∗∗	∗∗	∗	0	∗∗	7

This table identifies “high” quality choices with a “star.” A study can be awarded a maximum of one star for each numbered item within the selection and exposure categories. A maximum of 2 stars can be given for comparability.

**Table 2:** Characteristics of case-control studies on SEPP1 polymorphisms and cancer risk included in the meta-analysis.

Variants	Author	Year	Tumor type	Ethnicity	Genotyping method	Source of controls	Case			Control			
							GG	GA	AA	GG	GA	AA	*P* (HWE)
rs7579	Méplan et al.	2013	BC	European	TaqMan	PB	455	396	86	436	420	101	0.992
	Steinbrecher et al.	2010	PCa	European	MALDI-TOF	PB	116	105	27	250	209	33	0.224
	Méplan et al.	2010	CRC	European	KASPar	HB	260	369	61	269	323	37	*≤0.001*
	Sutherland et al.	2010	CRC	Asian	PCR-RFLP	HB	190	121	32	363	239	67	*0.004*
	Amini et al.	2019	CRC	Asian	PCR-HRM	PB	24	22	14	40	23	11	*0.022*

rs3877899	Steinbrecher et al.	2010	PCa	European	MALDI-TOF	PB	152	86	10	271	194	27	0.309
	Cooper et al.	2008	PCa	European	TaqMan	PB	1522	949	172	878	595	97	0.775
	Geybels et al.	2013	PCa	European	MALDI-TOF	PB	758	441	53	739	426	67	0.585
	Karunasinghe et al.	2012	PCa	European	TaqMan	PB	153	88	18	255	162	19	0.286
	Méplan et al.	2010	CRC	European	KASPar	HB	427	258	47	409	204	44	*0.009*
	Sutherland et al.	2010	CRC	Asian	PCR-RFLP	HB	797	5	0	710	1	0	0.985
	Méplan et al.	2013	BC	European	TaqMan	PB	586	321	30	594	317	48	0.499
	Jablonska et al.	2015	BC	European	TaqMan	PB	81	44	9	122	55	6	0.948
	Mohammaddoust et al.	2017	BC	Asian	PCR-RFLP	PB	80	37	33	151	33	16	*≤0.001*

BC: breast cancer; PCa: prostate cancer; CRC: colorectal cancer; PB: population-based; HB: hospital-based; MALDI-TOF: matrix-assisted laser desorption/ionizing time-of-flight mass spectrometry; PCR-RFLP: polymerase chain reaction-restriction fragment length polymorphism; PCR-HRM: polymerase chain reaction high-resolution melting method; KASPar: KBiosciences' Competitive Allele-Specific PCR; TaqMan: TaqMan fluorescent probe method.

**Table 3:** Subgroup analyses of the SEPP1 polymorphisms and cancer risk.

Variants	Comparison	Subgroup	*N*	*P* value			Regression model	
				*P*_H_	*P*_Z_	*P*_E_	Random	Fixed
rs3877899	A vs. G	Overall	9	≤0.001	0.243	0.104	1.099 (0.938-1.287)	1.008 (0.948-1.071)
	A vs. G	PCa	4	0.514	0.206		0.954 (0.885-1.027)	0.953 (0.885-1.027)
	A vs. G	BC	3	≤0.001	0.240		1.480 (0.770-2.844)	1.134 (0.992-1.295)
	A vs. G	PB	7	≤0.001	0.335		1.093 (0.912-1.310)	0.993 (0.931-1.059)
	A vs. G	Y	7	0.293	0.231		0.962 (0.888-1.042)	0.961 (0.899-1.026)
	A vs. G	European	7	0.269	0.434		0.979 (0.910-1.055)	0.976 (0.917-1.038)
	AA vs. GG	Overall	8	≤0.001	0.498	0.337	1.129 (0.794-1.605)	1.021 (0.871-1.196)
	AA vs. GG	PCa	4	0.206	0.604		0.946 (0.718-1.246)	0.949 (0.780-1.155)
	AA vs. GG	BC	3	≤0.001	0.405		1.726 (0.478-6.237)	1.269 (0.902-1.784)
	AA vs. GG	PB	7	≤0.001	0.483		1.162 (0.765-1.765)	1.020 (0.861-1.210)
	AA vs. GG	Y	6	0.081	0.599		0.925 (0.693-1.236)	0.917 (0.767-1.095)
	AA vs. GG	European	7	0.124	0.397		0.936 (0.738-1.187)	0.931 (0.790-1.098)
	AG vs. GG	Overall	9	0.036	0.603	0.109	1.035 (0.909-1.179)	1.003 (0.928-1.084)
	AG vs. GG	PCa	4	0.585	0.156		0.934 (0.850-1.027)	0.934 (0.850-1.026)
	AG vs. GG	BC	3	0.046	0.203		1.300 (0.868-1.947)	1.123 (0.948-1.331)
	AG vs. GG	PB	7	0.072	0.975		0.998 (0.875-1.138)	0.976 (0.898-1.060)
	AG vs. GG	Y	7	0.478	0.351		0.960 (0.883-1.044)	0.961 (0.884-1.045)
	AG vs. GG	European	7	0.308	0.703		0.989 (0.904-1.082)	0.985 (0.911-1.065)
	AG+AA vs. GG	Overall	9	0.001	0.356	0.098	1.079 (0.919-1.267)	1.008 (0.936-1.085)
	AG+AA vs. GG	PCa	4	0.623	0.152		0.936 (0.855-1.025)	0.936 (0.855-1.025)
	AG+AA vs. GG	BC	3	≤0.001	0.210		1.474 (0.804-2.704)	1.150 (0.980-1.350)
	AG+AA vs. GG	PB	7	0.001	0.551		1.056 (0.884-1.261)	0.984 (0.909-1.064)
	AG+AA vs. GG	Y	7	0.452	0.266		0.955 (0.881-1.034)	0.956 (0.882-1.035)
	AG+AA vs. GG	European	7	0.317	0.553		0.981 (0.901-1.068)	0.978 (0.907-1.054)
	AA vs. AG+GG	Overall	8	0.001	0.555	0.371	1.103 (0.797-1.526)	1.017 (0.871-1.189)
	AA vs. AG+GG	PCa	4	0.176	0.811		0.974 (0.733-1.295)	0.977 (0.806-1.184)
	AA vs. AG+GG	BC	3	≤0.001	0.445		1.580 (0.489-5.108)	1.199 (0.856-1.681)
	AA vs. AG+GG	PB	7	0.001	0.497		1.143 (0.777-1.681)	1.027 (0.869-1.215)
	AA vs. AG+GG	Y	6	0.067	0.699		0.944 (0.704-1.265)	0.936 (0.786-1.115)
	AA vs. AG+GG	European	7	0.113	0.444		0.940 (0.741-1.192)	0.939 (0.799-1.103)

rs7579	A vs. G	Overall	5	0.015	0.309	0.233	1.090 (0.923-1.286)	1.044 (0.958-1.137)
	A vs. G	CRC	3	0.079	0.236		1.148 (0.914-1.443)	1.125 (0.995-1.273)
	A vs. G	European	3	0.013	0.449		1.084 (0.880-1.335)	1.047 (0.952-1.152)
	A vs. G	PB	3	0.015	0.405		1.137 (0.840-1.539)	1.01 (0.892-1.123)
	A vs. G	N	3	0.079	0.236		1.148 (0.914-1.443)	1.125 (0.995-1.273)
	AA vs. GG	Overall	5	0.014	0.230	0.157	1.267 (0.861-1.866)	1.128 (0.923-1.379)
	AA vs. GG	CRC	3	0.091	0.204		1.387 (0.837-2.296)	1.329 (0.989-1.786)
	AA vs. GG	European	3	0.007	0.337		1.310 (0.755-2.274)	1.147 (0.910-1.445)
	AA vs. GG	PB	3	0.019	0.383		1.334 (0.698-2.548)	1.043 (0.802-1.356)
	AA vs. GG	N	3	0.091	0.204		1.387 (0.837-2.296)	1.329 (0.989-1.786)
	AG vs. GG	Overall	5	0.315	0.715	0.264	1.029 (0.900-1.177)	1.022 (0.908-1.151)
	AG vs. GG	CRC	3	0.357	0.223		1.112 (0.932-1.327)	1.112 (0.937-1.321)
	AG vs. GG	European	3	0.190	0.753		1.034 (0.866-1.234)	1.022 (0.895-1.166)
	AG vs. GG	PB	3	0.276	0.693		0.994 (0.808-1.224)	0.968 (0.825-1.136)
	AG vs. GG	N	3	0.357	0.223		1.112 (0.932-1.327)	1.112 (0.937-1.321)
	AG+AA vs. GG	Overall	5	0.071	0.434	0.210	1.075 (0.897-1.287)	1.038 (0.927-1.162)
	AG+AA vs. GG	CRC	3	0.149	0.116		1.158 (0.897-1.496)	1.140 (0.968-1.342)
	AG+AA vs. GG	European	3	0.051	0.551		1.072 (0.852-1.350)	1.039 (0.915-1.180)
	AG+AA vs. GG	PB	3	0.069	0.581		1.093 (0.798-1.497)	0.983 (0.845-1.144)
	AG+AA vs. GG	N	3	0.149	0.116		1.158 (0.897-1.287)	1.140 (0.968-1.342)
	AA vs. AG+GG	Overall	5	0.053	0.245	0.180	1.209 (0.878-1.666)	1.117 (0.921-1.355)
	AA vs. AG+GG	CRC	3	0.189	0.109		1.280 (0.861-1.903)	1.261 (0.949-1.676)
	AA vs. AG+GG	European	3	0.022	0.316		1.268 (0.797-2.019)	1.139 (0.912-1.422)
	AA vs. AG+GG	PB	3	0.046	0.396		1.264 (0.736-2.169)	1.054 (0.820-1.355)
	AA vs. AG+GG	N	3	0.189	0.109		1.280 (0.861-1.903)	1.261 (0.949-1.676)

PCa: prostate cancer; BC: breast cancer; CRC: colorectal cancer; HWE: Hardy-Weinberg Equilibrium; Y: study conformed to HWE; N: study did not conform to HWE; P-B: population-based; H-B: hospital-based; *P*_E_ = *P* value of Egger test; *P*_H_ = *P* value of heterogeneity test; *P*_Z_ = *P* value of Z test.

## Data Availability

The dataset can be accessed from the corresponding author upon reasonable request.

## Supplementary Materials

Supplementary Materials Table S1: scale for quality assessment.
