# Supplementary material for: Lack of Association between Common Polymorphisms in Selenoprotein P Gene and Susceptibility to Colorectal Cancer, Breast Cancer, and Prostate Cancer: A Meta-Analysis
Source: Biomed Res Int. 2021 Sep 27;2021:6525449. doi: 10.1155/2021/6525449 (PMC8490044; doi:10.1155/2021/6525449)
Supplement: Supplementary Materials — Table S1: scale for quality assessment. [file 6525449.f1.docx]

**Table S1.** Scale for quality assessment.

| **Criteria** | **Score** |
| --- | --- |
| **Representativeness of cases** |  |
| Consecutive/randomly selected form case population with clearly defined sampling frame | 2 |
| Consecutive/randomly selected form case population without clearly defined sampling frame or with extensive | 1 |
| Not described | 0 |
| **Source of controls** |  |
| Population- or Hospital-bases | 2 |
| Hospital-bases | 1 |
| Not described | 0 |
| **Hardy-Weinberg equilibrium in controls** |  |
| Hardy-Weinberg equilibrium | 2 |
| Hardy-Weinberg disequilibrium | 1 |
| **Genotyping examination** |  |
| Genotyping done under “blinded” condition | 1 |
| Unblended done or not mentioned | 0 |
| **Association assessment** |  |
| Assess association between genotypes and head and cancer with appropriate statistics and adjustment for confounders | 2 |
| Assess association between genotypes and head and cancer with appropriate statistics and without adjustment for confounders | 1 |
| Inappropriate statistics used | 0 |
